# Training safer surgeons: How do patients view the role of simulation in orthopaedic training?

**DOI:** 10.1186/s13037-015-0058-5

**Published:** 2015-03-07

**Authors:** Kashif Akhtar, Kapil Sugand, Asanka Wijendra, Nigel J Standfield, Justin P Cobb, Chinmay M Gupte

**Affiliations:** MSk Lab, Imperial College London, Charing Cross Hospital, London, , W6 8RF, , UK; Postgraduate School of Surgery, London Deanery, Stewart House, 32 Russell Square, London, , WC1B 5DN, , UK

**Keywords:** *Public perception*, *Surgical simulation*, *Perception*, *Arthroscopy*, *Virtual reality*

## Abstract

**Background:**

Simulation allows training without posing risk to patient safety. It has developed in response to the demand for patient safety and the reduced training times for surgeons. Whilst there is an increasing role of simulation in orthopaedic training, the perception of patients and the general public of this novel method is yet unknown. Patients and the public were given the opportunity to perform a diagnostic knee arthroscopy on a virtual reality ARTHRO Mentor simulator. After their practice session, participants answered a validated questionnaire based on a 5-point Likert Scale assessing their opinions on arthroscopic simulation. Primary objective was observing perception of patients on orthopaedic virtual reality simulation.

**Findings:**

There were a total of 159 respondents, of which 86% were of the opinion that simulators are widely used in surgical training and 94% felt that they should be compulsory. 91% would feel safer having an operation by a surgeon trained on simulators, 87% desired their surgeon to be trained on simulators and 72% believed that additional simulator training resulted in better surgeons. Moreover, none of the respondents would want their operation to be performed by a surgeon who had not trained on a simulator. Cronbach’s alpha was 0.969.

**Conclusions:**

There is also a clear public consensus for this method of training to be more widely utilised and it would enhance public perception of safer training of orthopaedic surgeons. This study of public perception provides a mandate to increase investment and infrastructure in orthopaedic simulation as part of promoting clinical governance.

## Background

### Current challenges

Enforced changes in postgraduate medical education are resulting in doctors attaining less experience than their predecessors. The European Working Time Directive (EWTD), changes to working practices and an increasing focus on patient safety have resulted in a significant reduction in the experience of surgical trainees [1]. This has implications for safety and training, particularly in craft surgical specialties where it has traditionally been a case of ‘practice makes perfect’. Training has become more challenging as surgical procedures increase in complexity with newer minimally invasive and computer-assisted approaches that have their own inherent learning curves. Trainers may also be reluctant to let trainees operate as independently as in previous generations in light of growing medico-legal claims, with more than £800 million of compensation paid out annually to patients by the NHS [2,3].

### The role of simulation

Medical educational and training has developed in an attempt to meet the current challenges. The role of simulation has increased significantly over the previous decade, with Simulated Patients (SP) and models [4] used in the re-enactment of a clinical setting within a controlled environment to learn and practise skills which can either be technical or non-technical (e.g. communication, leadership and teamwork). The aim is to demonstrate competence and confidence as well as reducing the risk of error when operating on patients.

Simulation offers a safe environment in which to augment psychomotor skills in a controlled and efficient manner without posing a risk to patients or to learners [5-7]. The Chief Medical Officer’s Annual Report in 2008, Sir Liam Donaldson stated that *“Simulation*-*based training should be fully integrated and funded within training programmes for clinicians at all stages”* [3]. The General Medical Council (GMC) has also stressed the importance of simulation in training, whilst the Food and Drug Administration (FDA) stated, as far back as year 2004, that simulation should be an important part of any carotid artery stenting programme [8]. Trainees can hone their surgical skills safely, free of time and service pressures.

### Virtual reality simulators

Much work has been done on the use of Virtual Reality (VR) simulators in laparoscopic surgery where skills learnt using the simulators have been shown to cross over into the clinical setting. This has been shown to shorten the learning curve on real laparoscopic procedures and to significantly reduce errors in live surgery [9-13]. This ‘crossover’ benefit of simulation has been proven repeatedly in laparoscopic surgery [14]. Simulation can provide objective data on economy of movement, time taken and collateral damage to anatomical structures. It can provide a means of repeated practice and facilitate self-directed learning at a pace appropriate for each individual. It has also recently been used in the selection of surgical trainees and career progression. The role of VR simulators in orthopaedic surgery is gradually increasing but it is still very much in its infancy with little evidence-based research available within current literature.

### Public involvement

Healthcare is a public service paid for by citizens. Patient and public involvement is one of the pillars of clinical governance and there is a growing emphasis on patient choice over treatment and care, as well as involving patients in decisions about health care provision and the monitoring of outcomes. There has been a proliferation of questionnaires, interview schedules and rating scales to record measures of health and illness from the patient’s point of view. Patient-Reported Outcome Measures (PROMs) assess the quality of care delivered to NHS patients from the patient perspective. PROMS have been collected by all providers of NHS-funded care since April 2009.

Patient education offers empowerment and ownership. The public is increasingly involved in developing and improving healthcare services and is represented by surveys on behalf of the Health Commission, Local Involvement Networks or Patient Forums, and as Lay Members on the Foundation Trust of Board of Governors.

With respect to surgery, public perception of surgical operations is often of structured and controlled events, where high levels of skill combined with clinical detachment are expected [15]. Furthermore, public opinion is resistant to patients being used as training material, especially if there is a compromise to patient safety. Simulation provides the opportunity to learn and rehearse operations in a safe, controlled and measurable environment [16] under appropriate supervision. Whilst there is public awareness of the role of simulators in the training of airline pilots, there is no published literature on the public and patient perception of simulation in surgical training.

## Methods

At a public exhibition at the Science Museum of London, a high fidelity VR simulator (ARTHRO Mentor™, Simbionix, Ohio, USA) was made available for members of the public to use and perform diagnostic knee arthroscopy. A questionnaire was designed to gauge public opinion on the role of simulators in surgical training, based on a five-point Likert scale. 159 participants fully completed the questionnaire, which was initially validated by two independent consultant orthopaedic surgeons and two members of the public. Internal validity of the questionnaire was calculated using Cronbach’s alpha. All data were analysed using Microsoft Excel (Microsoft, New York, USA). Ethics was granted by Imperial College Medical Education Ethics Committee (MEEC1213-17).

### Findings

As seen in Table 1, all those taking part enjoyed using the simulator and 91% felt better informed about arthroscopy. 86% of people assumed that simulators are widely used in surgical training and 94% felt that they should be compulsory. 91% would feel safer having an operation by a surgeon trained on simulators, 87% desired their surgeon to be trained on simulators and 72% believed that additional simulator training resulted in better surgeons.

**Table 1:** **Results of the public questionnaire** (**percentage** - %)

***Question***	**Strongly Disagree**	**Disagree**	**Neither agree or disagree**	**Agree**	**Strongly Agree**
*1. Simulators are widely used in surgical training*	0	6	8	48	38
*2. Simulators should be compulsory in surgical training*	0	1	5	38	59
*3. I would feel safer having an operation by a surgeon who has trained with simulators*	0	1	8	28	63
*4. I believe that surgeons trained additionally on simulators are better surgeons*	0	3	26	24	47
*5. 1 wuold want my operation to be performed by a surgeon who has trained additionally with simulators*	0	0	13	31	56
*6. I enjoyed using the simulators*	0	0	0	33	67
*7. I feel better informed about arthroscopic surgery having used a simulator*	0	0	9	41	50

**Figure 1:**
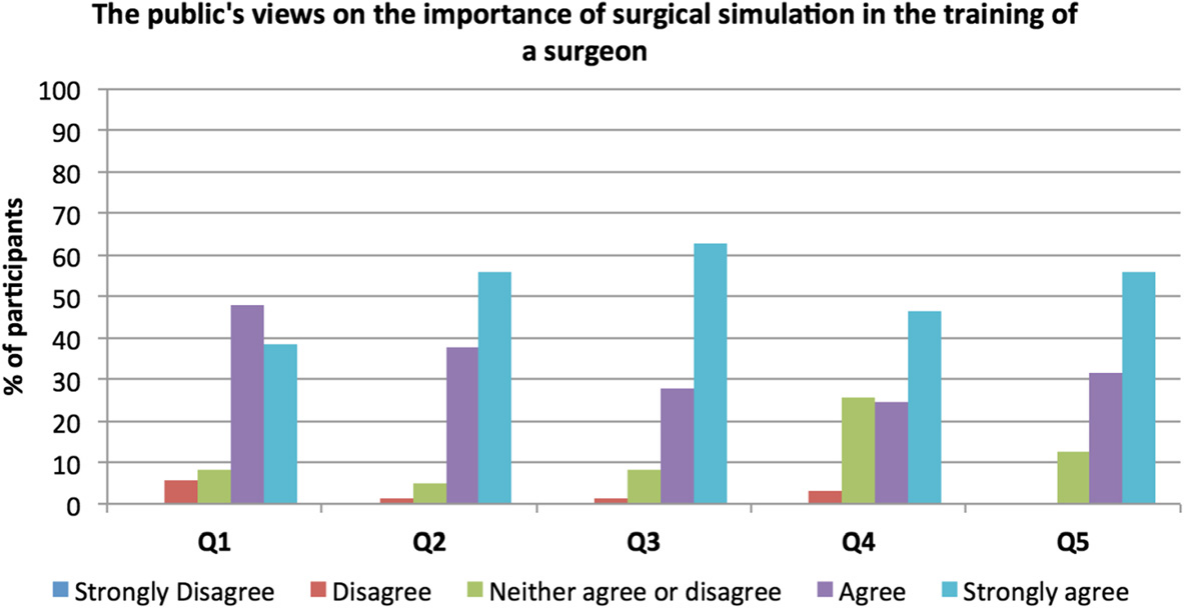
**Public perception of simulation in surgical training**.

Moreover, none of the respondents would want their operation to be performed by a surgeon who had not trained on a simulator. The internal validity of the questionnaire using Cronbach’s alpha was 0.969. Figure 1 displays visual comparisons between incidence of opinions for each question.

The role of simulation in educating surgeons is developing rapidly as a means of compensating for the significant changes to post-graduate training following the introduction of Modernising Medical Careers (MMC) and the EWTD. There is a need for doctors to gain experience in a safe, controlled environment away from service pressures. One way to address this is through simulation. Simulation provides a focused and effective way of repeatedly practising technical skills without risk to learner or patient. It permits standardised practice and can facilitate self-directed learning at a pace appropriate for each individual. VR simulation is playing an increasingly important role in surgical training as the quality and fidelity of simulators improve with time.

It is interesting that the vast majority of the public surveyed thought that simulators are widely used in surgical training when this is not the case, particularly in trauma and orthopaedic surgery. The overwhelming majority felt that simulators should be compulsory for trainee surgeons. There was also a very high internal validity of the questionnaire among all participants. Simulation may save much money in the long-run since there is significant cost in training surgical trainees in the operating theatre. This has previously been estimated in USA as $53 million a year [17].

Simulation may be heading towards becoming an integral part within both undergraduate and postgraduate training that can help to identify trainees with adequate psychomotor skills from an early stage to reduce risk of errors. Nevertheless, this concept has yet to be validated through further research and formally implemented. Albeit, there are some schools of surgery in the UK that have dedicated simulation centres and are supported by research grants, charities and deaneries. Within clinical practice, furthering education and patient empowerment are also major pillars of clinical governance and good medical practice.

## Conclusions

Simulation may mitigate risks to patients, practitioners and organisations. It is clear that the public wishes for surgeons to minimise their learning curves away from patients, in a safe environment. People want their surgeons to be safe, accountable and accredited. VR simulation can facilitate this and there is a desire for this to be more widely integrated into surgical training curriculum.

## Abbreviations

EWTD: European Working Time Directive
FDA: Food and Drug Administration
GMC: General Medical Council
MMC: Modernising Medical Careers
NHS: National Health Service
PROMs: Patient Reported Outcome Measures
SP: Simulated Patient
USA: United States of America
VR: Virtual Reality
